# Supplementary material for: Transcriptomics analysis reveals molecular alterations underpinning spaceflight dermatology
Source: Commun Med (Lond). 2024 Jun 11;4:106. doi: 10.1038/s43856-024-00532-9 (PMC11166967; doi:10.1038/s43856-024-00532-9)
Supplement: Supplementary file 2 — Supplementary information [file 43856_2024_532_MOESM2_ESM.pdf]

Supplementary Information

Transcriptomics Analysis Reveals Molecular Alterations Underpinning Spaceflight Dermatology

Henry Cope, Jonas Elsborg, Samuel Demharter, J. Tyson McDonald, Chiara Wernecke, Hari Parthasarathy, Hriday Unadkat, Mira Chatrathi, Jennifer Claudio, Sigrid Reinsch, Pinar Avci, Sara R. Zwart, Scott M. Smith, Martina Heer, Masafumi Muratani, Cem Meydan, Eliah Overbey, Jangkeun Kim, Christopher R. Chin, Jiwoon Park, Jonathan C. Schisler, Christopher E. Mason, Nathaniel J. Szewczyk, Craig R. G. Willis, Amr Salam, Afshin Beheshti

Supplementary Table 1. Curated pathways for directed pathway analysis.

Supplementary Figure 1. All cross-mission genes involved in rodent skin spaceflight response.

Supplementary Figure 2. The profile of all the cross-mission genes in astronauts.

Supplementary Figure 3. The profile of subset of the skin health genes in astronauts.

Supplementary Data 1. Differential Gene Expression Results.

Supplementary Data 2. Gene Set Enrichment Analysis Results.

Supplementary Data 3. Supplemental IPA Drug Results

Supplementary Data 4. Source Data for Figures

Supplementary Table 1. Curated pathways for directed pathway analysis. Pathway lists used in our analysis for **Figs. 5 – 7**.

Curated skin health pathways:

HP_ABNORMALITY_OF_SKIN_PHYSIOLOGY
HP_ABNORMALITY_OF_SKIN_ADNEXA_PHYSIOLOGY
HP_ABNORMALITY_OF_EPIDERMAL_MORPHOLOGY
GOBP_EPIDERMIS_MORPHOGENESIS
GOBP_REGULATION_OF_EPIDERMIS_DEVELOPMENT
GOBP_NEGATIVE_REGULATION_OF_EPIDERMIS_DEVELOPMENT
GOBP_REGULATION_OF_KERATINOCYTE_DIFFERENTIATION
GOMF_KERATIN_FILAMENT_BINDING
REACTOME_KERATINIZATION
REACTOME_COLLAGEN_BIOSYNTHESIS_AND_MODIFYING_ENZYMES
GOBP_ELASTIN_CATABOLIC_PROCESS
GOBP_ELASTIN_METABOLIC_PROCESS
HP_DERMAL_ATROPHY
HP_ABNORMAL_ELASTICITY_OF_SKIN
HP_THIN_SKIN
HP_THICKENED_SKIN
ENK_UV_RESPONSE_EPIDERMIS_DN
DURCHDEWALD_SKIN_CARCINOGENESIS_DN
ENK_UV_RESPONSE_KERATINOCYTE_DN
GOBP_REGULATION_OF_WATER_LOSS_VIA_SKIN

Curated DNA damage & repair pathways:

Pathway	Category
GOBP_BASE_EXCISION_REPAIR	Base Excision Repair
GOBP_BASE_EXCISION_REPAIR_AP_SITE_FORMATION	Base Excision Repair
GOBP_BASE_EXCISION_REPAIR_GAP_FILLING	Base Excision Repair
KEGG_BASE_EXCISION_REPAIR	Base Excision Repair
REACTOME_BASE_EXCISION_REPAIR	Base Excision Repair
REACTOME_BASE_EXCISION_REPAIR_AP_SITE_FORMATION	Base Excision Repair
REACTOME_DISEASES_OF_BASE_EXCISION_REPAIR	Base Excision Repair
REACTOME_PCNA_DEPENDENT_LONG_PATCH_BASE_EXCISION_REPAIR	Base Excision Repair
REACTOME_POLB_DEPENDENT_LONG_PATCH_BASE_EXCISION_REPAIR	Base Excision Repair
WP_BASE_EXCISION_REPAIR	Base Excision Repair
GOBP_CELLULAR_RESPONSE_TO_DNA_DAMAGE_STIMULUS	DDR
GOBP_DNA_DAMAGE_INDUCED_PROTEIN_PHOSPHORYLATION	DDR
GOBP_DNA_DAMAGE_RESPONSE_DETECTION_OF_DNA_DAMAGE	DDR
GOBP_DNA_DAMAGE_RESPONSE_SIGNAL_TRANSDUCTION_BY_P53_CLASS_MEDIATOR	DDR
GOBP_DNA_DAMAGE_RESPONSE_SIGNAL_TRANSDUCTION_RESULTING_IN_TRANSCRIPTION	DDR
GOBP_G2_DNA_DAMAGE_CHECKPOINT	DDR
GOBP_INTRA_S_DNA_DAMAGE_CHECKPOINT	DDR
GOBP_INTRINSIC_APOPTOTIC_SIGNALING_PATHWAY_IN_RESPONSE_TO_DNA_DAMAGE	DDR
GOBP_INTRINSIC_APOPTOTIC_SIGNALING_PATHWAY_IN_RESPONSE_TO_DNA_DAMAGE_BY_P53_CLASS_MEDIATOR	DDR
GOBP_MITOTIC_G2_DNA_DAMAGE_CHECKPOINT	DDR
GOBP_NEGATIVE_REGULATION_OF_DNA_DAMAGE_CHECKPOINT	DDR
GOBP_NEGATIVE_REGULATION_OF_DNA_DAMAGE_RESPONSE_SIGNAL_TRANSDUCTION_BY_P53_CLASS_MEDIATOR	DDR
GOBP_NEGATIVE_REGULATION_OF_INTRINSIC_APOPTOTIC_SIGNALING_PATHWAY_IN_RESPONSE_TO_DNA_DAMAGE	DDR
GOBP_NEGATIVE_REGULATION_OF_RESPONSE_TO_DNA_DAMAGE_STIMULUS	DDR
GOBP_POSITIVE_REGULATION_OF_INTRINSIC_APOPTOTIC_SIGNALING_PATHWAY_IN_RESPONSE_TO_DNA_DAMAGE	DDR
GOBP_POSITIVE_REGULATION_OF_RESPONSE_TO_DNA_DAMAGE_STIMULUS	DDR
GOBP_PRE_REPLICATIVE_COMPLEX_ASSEMBLY_INVOLVED_IN_CELL_CYCLE_DNA_REPLICATION	DDR
GOBP_REGULATION_OF_DNA_DAMAGE_CHECKPOINT	DDR
GOBP_REGULATION_OF_DNA_DAMAGE_RESPONSE_SIGNAL_TRANSDUCTION_BY_P53_CLASS_MEDIATOR	DDR
GOBP_REGULATION_OF_INTRINSIC_APOPTOTIC_SIGNALING_PATHWAY_IN_RESPONSE_TO_DNA_DAMAGE	DDR
GOBP_REGULATION_OF_INTRINSIC_APOPTOTIC_SIGNALING_PATHWAY_IN_RESPONSE_TO_DNA_DAMAGE_BY_P53_CLASS_MEDIATOR	DDR
GOBP_REGULATION_OF_RESPONSE_TO_DNA_DAMAGE_STIMULUS	DDR
GOBP_SIGNAL_TRANSDUCTION_IN_RESPONSE_TO_DNA_DAMAGE	DDR
GOBP_SIGNAL_TRANSDUCTION_INVOLVED_IN_G2_DNA_DAMAGE_CHECKPOINT	DDR
GOBP_TELOMERE_MAINTENANCE_IN_RESPONSE_TO_DNA_DAMAGE	DDR
GOBP_UV_DAMAGE_EXCISION_REPAIR	DDR
GOCC_SITE_OF_DNA_DAMAGE	DDR
GOMF_DAMAGED_DNA_BINDING	DDR
KYNG_DNA_DAMAGE_BY_4NQO	DDR
KYNG_DNA_DAMAGE_BY_4NQO_OR_GAMMA_RADIATION	DDR
KYNG_DNA_DAMAGE_BY_4NQO_OR_UV	DDR
KYNG_DNA_DAMAGE_BY_GAMMA_AND_UV_RADIATION	DDR
KYNG_DNA_DAMAGE_BY_GAMMA_RADIATION	DDR
KYNG_DNA_DAMAGE_BY_UV	DDR
KYNG_DNA_DAMAGE_DN	DDR

KYNG_DNA_DAMAGE_UP	DDR
REACTOME_DNA_DAMAGE_BYPASS	DDR
REACTOME_DNA_DAMAGE_REVERSAL	DDR
REACTOME_DNA_DAMAGE_TELOMERE_STRESS_INDUCED_SENESCENCE	DDR
REACTOME_G1_S_DNA_DAMAGE_CHECKPOINTS	DDR
REACTOME_G2_M_DNA_DAMAGE_CHECKPOINT	DDR
REACTOME_INHIBITION_OF_REPLICATION_INITIATION_OF_DAMAGED_DNA_BY_RB1_E2F1	DDR
REACTOME_RECOGNITION_OF_DNA_DAMAGE_BY_PCNA_CONTAINING_REPLICATION_COMPLEX	DDR
REACTOME_SUMOYLATION_OF_DNA_DAMAGE_RESPONSE_AND_REPAIR_PROTEINS	DDR
REACTOME_TP53_REGULATES_TRANSCRIPTION_OF_DNA_REPAIR_GENES	DDR
WP_DNA_DAMAGE_RESPONSE	DDR
WP_DNA_DAMAGE_RESPONSE_ONLY_ATM_DEPENDENT	DDR
WP_DNA_IRDAMAGE_AND_CELLULAR_RESPONSE_VIA_ATR	DDR
WP_MIRNA_REGULATION_OF_DNA_DAMAGE_RESPONSE	DDR
WP_MIRNAS_INVOLVED_IN_DNA_DAMAGE_RESPONSE	DDR
GOBP_DNA_DOUBLE_STRAND_BREAK_PROCESSING	DNA DSB
GOBP_DOUBLE_STRAND_BREAK_REPAIR	DNA DSB
GOBP_DOUBLE_STRAND_BREAK_REPAIR_INVOLVED_IN_MEIOTIC_RECOMBINATION	DNA DSB
GOBP_DOUBLE_STRAND_BREAK_REPAIR_VIA_BREAK_INDUCED_REPLICATION	DNA DSB
GOBP_DOUBLE_STRAND_BREAK_REPAIR_VIA_CLASSICAL_NONHOMOLOGOUS_END_JOINING	DNA DSB
GOBP_DOUBLE_STRAND_BREAK_REPAIR_VIA_SINGLE_STRAND_ANNEALING	DNA DSB
GOBP_DOUBLE_STRAND_BREAK_REPAIR_VIA_SYNTHESIS_DEPENDENT_STRAND_ANNEALING	DNA DSB
GOBP_MEIOTIC_DNA_DOUBLE_STRAND_BREAK_FORMATION	DNA DSB
GOBP_NEGATIVE_REGULATION_OF_DOUBLE_STRAND_BREAK_REPAIR_VIA_HOMOLOGOUS_RECOMBINATION	DNA DSB
GOBP_NEGATIVE_REGULATION_OF_DOUBLE_STRAND_BREAK_REPAIR_VIA_NONHOMOLOGOUS_END_JOINING	DNA DSB
GOBP_NON_RECOMBINATIONAL_REPAIR	DNA DSB
GOBP_POSITIVE_REGULATION_OF_DOUBLE_STRAND_BREAK_REPAIR	DNA DSB
GOBP_POSITIVE_REGULATION_OF_DOUBLE_STRAND_BREAK_REPAIR_VIA_HOMOLOGOUS_RECOMBINATION	DNA DSB
GOBP_POSITIVE_REGULATION_OF_DOUBLE_STRAND_BREAK_REPAIR_VIA_NONHOMOLOGOUS_END_JOINING	DNA DSB
GOBP_REGULATION_OF_DOUBLE_STRAND_BREAK_REPAIR	DNA DSB
GOBP_REGULATION_OF_DOUBLE_STRAND_BREAK_REPAIR_VIA_HOMOLOGOUS_RECOMBINATION	DNA DSB
GOBP_REGULATION_OF_DOUBLE_STRAND_BREAK_REPAIR_VIA_NONHOMOLOGOUS_END_JOINING	DNA DSB
GOBP_REPLICATION_BORN_DOUBLE_STRAND_BREAK_REPAIR_VIA_SISTER_CHROMATID_EXCHANGE	DNA DSB
GOMF_DOUBLE_STRANDED_METHYLATED_DNA_BINDING	DNA DSB
GOMF_DOUBLE_STRANDED_TELOMERIC_DNA_BINDING	DNA DSB
REACTOME_DNA_DOUBLE_STRAND_BREAK_REPAIR	DNA DSB
REACTOME_DNA_DOUBLE_STRAND_BREAK_RESPONSE	DNA DSB
REACTOME_HOMOLOGY_DIRECTED_REPAIR	DNA DSB
REACTOME_PROCESSING_OF_DNA_DOUBLE_STRAND_BREAK_ENDS	DNA DSB
REACTOME_SENSING_OF_DNA_DOUBLE_STRAND_BREAKS	DNA DSB
WP_DNA_IRDOUBLE_STRAND_BREAKS_DSBS_AND_CELLULAR_RESPONSE_VIA_ATM	DNA DSB
GOBP_DNA_DEALKYLATION_INVOLVED_IN_DNA_REPAIR	DNA Repair
GOBP_DNA_LIGATION_INVOLVED_IN_DNA_REPAIR	DNA Repair
GOBP_DNA_REPAIR	DNA Repair
GOBP_MITOCHONDRIAL_DNA_REPAIR	DNA Repair
GOBP_NEGATIVE_REGULATION_OF_DNA_REPAIR	DNA Repair
GOBP_POSITIVE_REGULATION_OF_DNA_REPAIR	DNA Repair
GOBP_REGULATION_OF_DNA_REPAIR	DNA Repair
GOCC_DNA_REPAIR_COMPLEX	DNA Repair
HP_ABNORMALITY_OF_DNA_REPAIR	DNA Repair
HP_DEFECTIVE_DNA_REPAIR_AFTER_ULTRAVIOLET_RADIATION_DAMAGE	DNA Repair
KAUFFMANN_DNA_REPAIR_GENES	DNA Repair
REACTOME_DISEASES_OF_DNA_REPAIR	DNA Repair
REACTOME_DNA_REPAIR	DNA Repair
WP_DNA_REPAIR_PATHWAYS_FULL_NETWORK	DNA Repair
GOBP_NEGATIVE_REGULATION_OF_SINGLE_STRANDED_VIRAL_RNA_REPLICATION_VIA_DOUBLE_STRANDED_DNA_INTERMEDIATE	DNA SSB
GOBP_SINGLE_STRAND_BREAK_REPAIR	DNA SSB
GOBP_MEIOTIC_MISMATCH_REPAIR	Mismatch Repair
GOBP_MISMATCH_REPAIR	Mismatch Repair
GOCC_MISMATCH_REPAIR_COMPLEX	Mismatch Repair
GOMF_MISMATCH_REPAIR_COMPLEX_BINDING	Mismatch Repair
GOMF_MISMATCHED_DNA_BINDING	Mismatch Repair
KEGG_MISMATCH_REPAIR	Mismatch Repair
REACTOME_DISEASES_OF_MISMATCH_REPAIR_MMR	Mismatch Repair
REACTOME_MISMATCH_REPAIR	Mismatch Repair
WP_DNA_MISMATCH_REPAIR	Mismatch Repair
GOBP_GLOBAL_GENOME_NUCLEOTIDE_EXCISION_REPAIR	Nucelotide Excision Repair
GOBP_NUCLEOTIDE_EXCISION_REPAIR	Nucelotide Excision Repair
GOBP_NUCLEOTIDE_EXCISION_REPAIR_DNA_DAMAGE_RECOGNITION	Nucelotide Excision Repair
GOBP_NUCLEOTIDE_EXCISION_REPAIR_DNA_DUPLEX_UNWINDING	Nucelotide Excision Repair
GOBP_NUCLEOTIDE_EXCISION_REPAIR_DNA_GAP_FILLING	Nucelotide Excision Repair
GOBP_NUCLEOTIDE_EXCISION_REPAIR_DNA_INCISION	Nucelotide Excision Repair

GOBP_NUCLEOTIDE_EXCISION_REPAIR_PREINCISION_COMPLEX_ASSEMBLY	Nucelotide Excision Repair
GOBP_NUCLEOTIDE_EXCISION_REPAIR_PREINCISION_COMPLEX_STABILIZATION	Nucelotide Excision Repair
GOBP_TRANSCRIPTION_COUPLED_NUCLEOTIDE_EXCISION_REPAIR	Nucelotide Excision Repair
GOCC_NUCLEOTIDE_EXCISION_REPAIR_COMPLEX	Nucelotide Excision Repair
KEGG_NUCLEOTIDE_EXCISION_REPAIR	Nucelotide Excision Repair
REACTOME_DNA_DAMAGE_RECOGNITION_IN_GG_NER	Nucelotide Excision Repair
REACTOME_GAP_FILLING_DNA_REPAIR_SYNTHESIS_AND_LIGATION_IN_GG_NER	Nucelotide Excision Repair
REACTOME_GLOBAL_GENOME_NUCLEOTIDE_EXCISION_REPAIR_GG_NER	Nucelotide Excision Repair
REACTOME_NUCLEOTIDE_EXCISION_REPAIR	Nucelotide Excision Repair
REACTOME_TRANSCRIPTION_COUPLED_NUCLEOTIDE_EXCISION_REPAIR_TC_NER	Nucelotide Excision Repair
WP_NUCLEOTIDE_EXCISION_REPAIR	Nucelotide Excision Repair

Curated/custom mitochondrial pathways:

Pathway	Genes
Folate Metabolism	*DHFR, SHMT1, MTHFR, MTR, MTRR, MTHFD1, ALDH1L2, MTFMT, DHFR2, SHMT2, GCLC (GCS), MTHFD2, MTHFD2L, GTPBP3/MT01, TYMS, ATIC, TK1, TK2, MTHFD1L*
Glutathione Synthesis	*GCLC, GCLM, GPX1, GPX2, GPX3, GSTA1, GSTM1, GSTO1, GSTP1, GSTZ1, GSTA4, GSTA2, GSTA3, GSTM2, GSTM3, GSTM4, GSTO2, GSTT2, GSTK1*
Glycolysis	*SLC2A1, SLC2A4, HK1, HK2, HK3, GCK, GPI, PFKM, PFKL, ALDOA, GAPDH, PGK1, ENO1, PKM, LDHA, LDHB, SLC16A1, SLC16A3, SLC16A4*
Selected HIF Target Genes	*VEGFA, EPO, PDGFB, PGF, CXCL12, CXCR4, HMOX1, ID2, IGF2, TERT, POU5F1, PGM1, TGFa, GADD45A, TGFb3, IGFBP3, NOS2, NOS3, PMAIP1, GPX3, SOD2, ADM, ANGPT1, ANGPT2, NPPA, ABCG2, CP, EDN1, FECH, KDR, FLT1, LEP, SERPINE1, TF, NT5E, CCN2, ENG, TFF3, MET, NR4A1, DDIT4, RORa, AURKA, WT1, SNAI1, SNAI2, TCF3, VIM, ZEB1, ZEB2, BNIP3, NDRG4, PPP5C, MCL1, NPM1, GPI, ANGPTL4, CTSC, L1CAM, LGALS1, LOX, LOXL2, LOXL4, MMP1, MMP2, MMP9, MMP14, PLAUR, STC2, TWIST1*
mTOR pathway	*TSC1, TSC2, RHEB, RPTOR, AK1S1, MTOR, DEPTOR, MLST8, TEL2, TTI1, CLIP-170, GRB10, LIPIN1, ATG1, EIF4EBP1, RPS6KB1, MAPKAP1, RICTOR, PRR5, PRR5L, PRKAA2, PRKAB1, PRKAG1, DDIT4, AKT1, AKT2, AKT3*
Peroxisome	*PEX1, PEX2, PEX3, PEX5, PEX6, PEX7, PEX10, PEX12, PEX13, PEX14, PEX26, PHYH, CAT, AGXT, ACOX1, HSD17B4, GNPAT, ABCD1, AMACR, AGPS, TRIM37*
ISR	*EIF2A, EIF2B2, EIF2AK1, EIF2AK2, EIF2AK3, EIF2AK4, ATF3, ATF4, ATF5, ATF6, DDIT3, PPP1R15A, FGF21, GDF15, XBP1, SESN2, SCAF1*

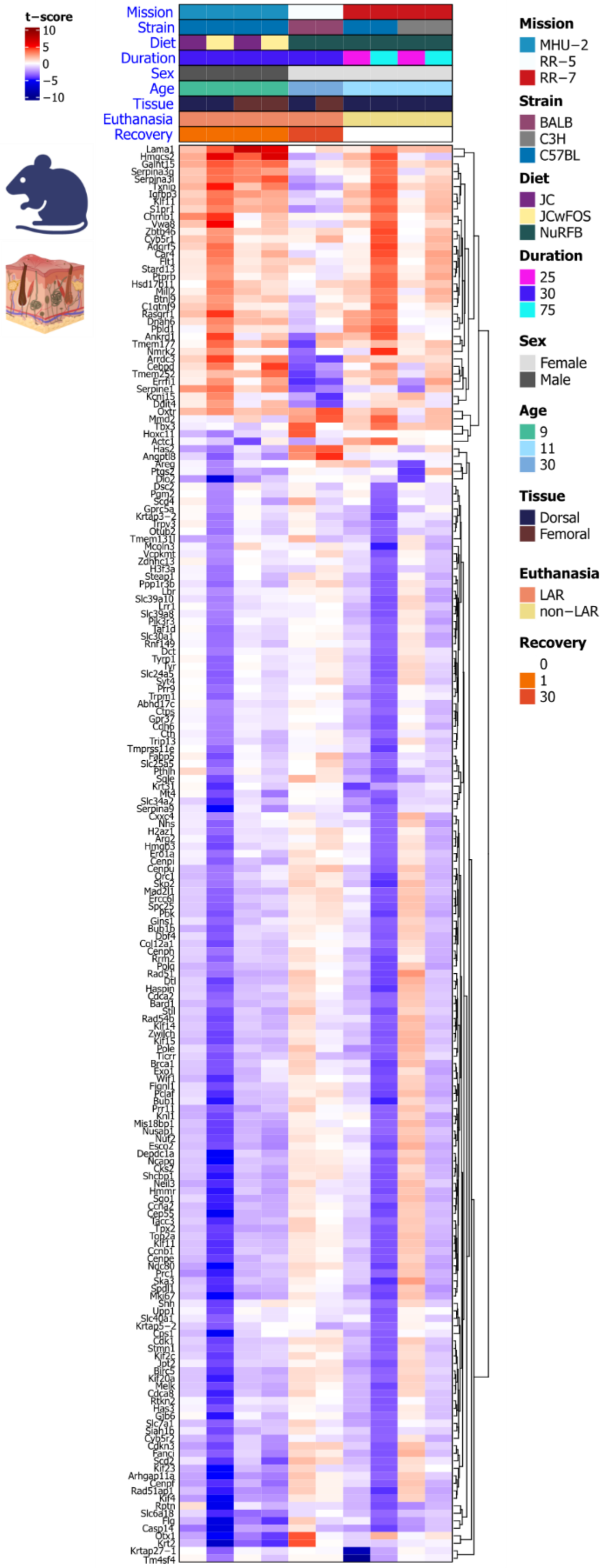

Supplementary Figure 1. All cross-mission genes involved in rodent skin spaceflight response. A heatmap showing regulatory changes of all cross-mission genes (genes that are significantly (FDR ≤ 0.1) differentially expressed between flight and ground control across multiple missions) within each rodent data subset.

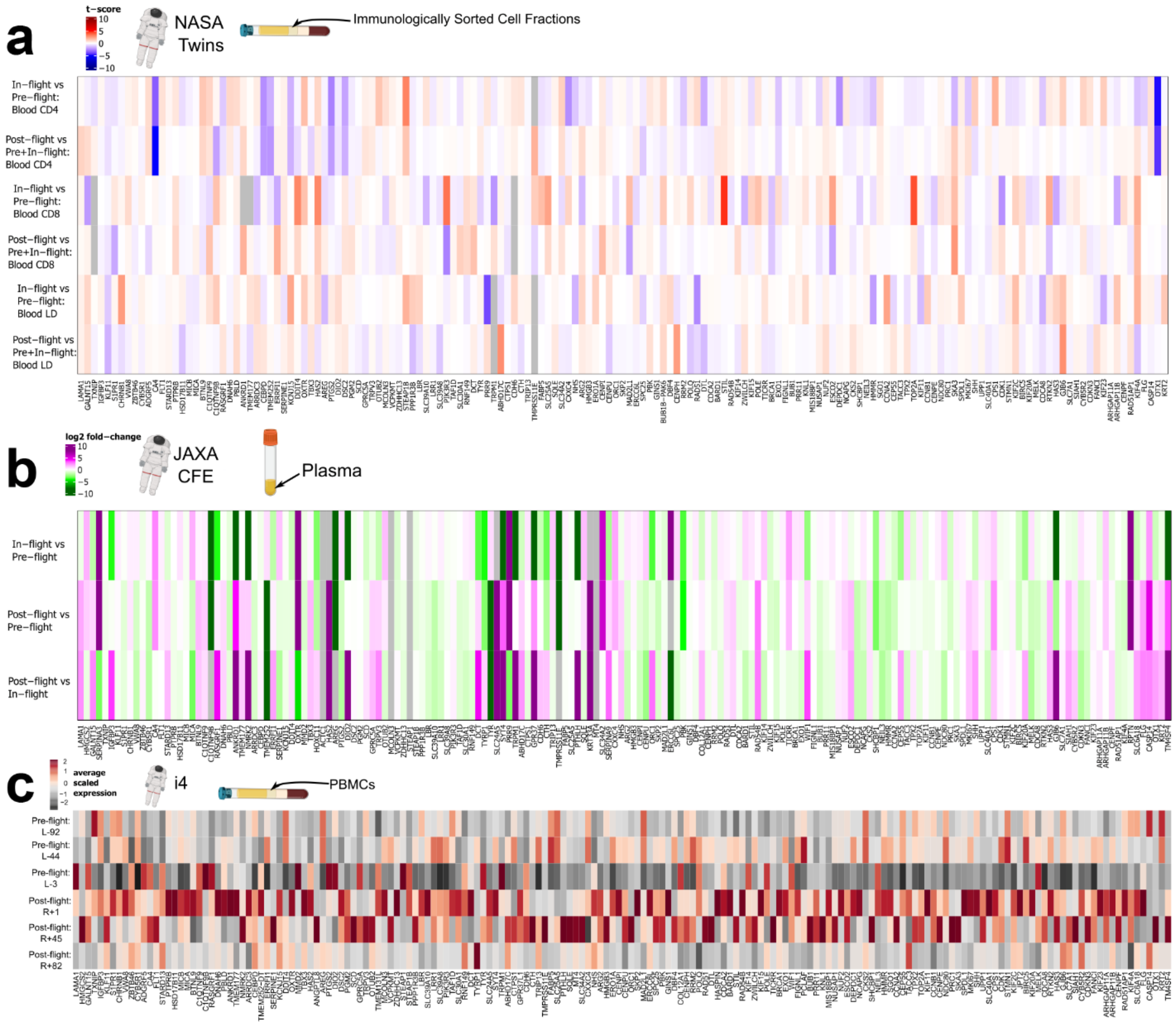

Supplementary Figure 2. The profile of all the cross-mission genes in astronauts. a) Heatmap showing t-score in orthologs of the rodent skin cross-mission genes in astronaut blood samples from sorted cell fractions from the NASA Twin Study. **b)** Heatmap showing $\log_2$ fold-change in orthologs of the rodent skin cross-mission genes in plasma samples at different time points from the JAXA CFE astronaut study. **c)** Heatmap showing average scaled expression in orthologs of the rodent skin cross-mission genes in astronaut PBMC data from the Inspiration4 mission at different timepoints.

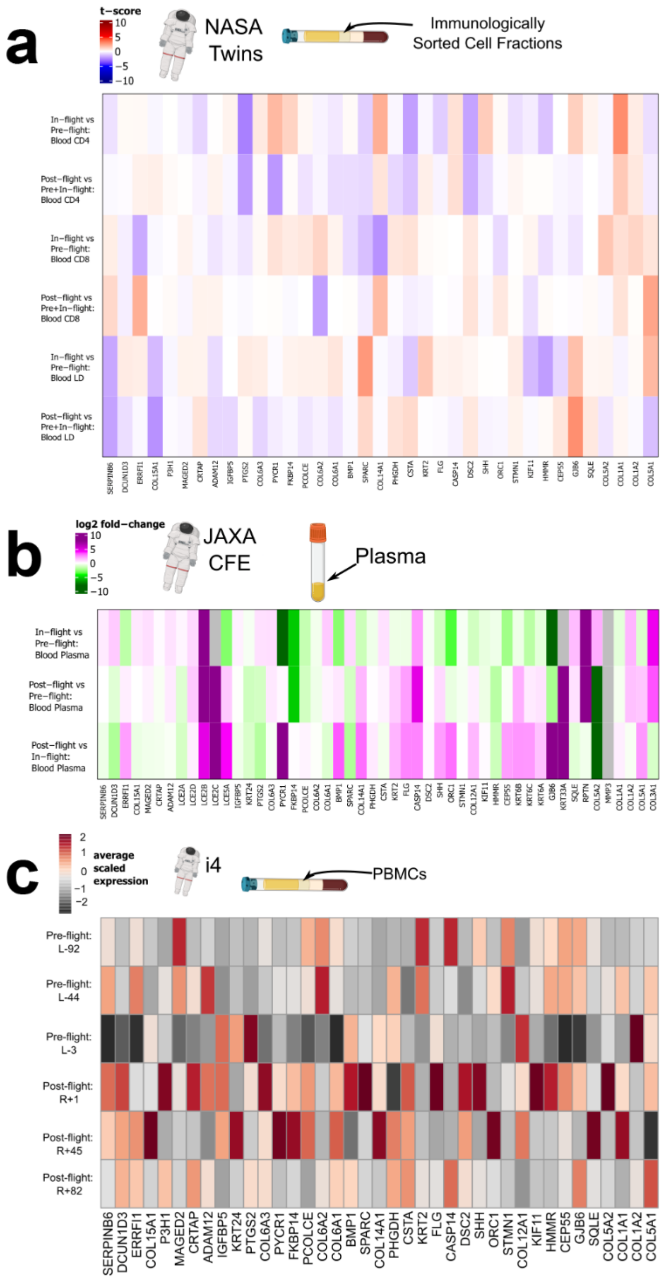

Supplementary Figure 3. The profile of subset of the skin health genes in astronauts. a) Heatmap showing t-score in orthologs of the rodent skin, skin health genes (from **Fig. 5**) in astronaut blood samples from sorted cell fractions from the NASA Twin Study. **b)** Heatmap showing log_2 fold-change in orthologs of the rodent skin, skin health genes (from **Fig. 5**) in plasma samples at different time points from the JAXA CFE astronaut study. **c)** Heatmap showing average scaled expression in orthologs of the rodent skin, skin health genes (from **Fig. 5**) in astronaut PBMC data from the Inspiration4 mission at different timepoints.
