# Supplementary material for: Transcriptomics analysis reveals molecular alterations underpinning spaceflight dermatology
Source: Commun Med (Lond). 2024 Jun 11;4:106. doi: 10.1038/s43856-024-00532-9 (PMC11166967; doi:10.1038/s43856-024-00532-9)
Supplement: Supplementary file 3 — Description of Additional Supplementary Files [file 43856_2024_532_MOESM3_ESM.pdf]

Description of Additional Supplementary Files

File name: Supplementary Data 1
Description: Differential Gene Expression Results

File name: Supplementary Data 2
Description: Gene Set Enrichment Analysis Results.

File name: Supplementary Data 3
Description: Supplemental IPA Drug Results
