# Supplementary material for: Transcriptomics analysis reveals molecular alterations underpinning spaceflight dermatology
Source: Commun Med (Lond). 2024 Jun 11;4:106. doi: 10.1038/s43856-024-00532-9 (PMC11166967; doi:10.1038/s43856-024-00532-9)
Supplement: Supplementary file 8 — Reporting Summary [file 43856_2024_532_MOESM8_ESM.pdf]

nature portfolio

Corresponding author(s): Afshin Beheshti

Last updated by author(s): Feb 21, 2024

Reporting Summary

Nature Portfolio wishes to improve the reproducibility of the work that we publish. This form provides structure for consistency and transparency in reporting. For further information on Nature Portfolio policies, see our Editorial Policies and the Editorial Policy Checklist.

Statistics

For all statistical analyses, confirm that the following items are present in the figure legend, table legend, main text, or Methods section.

n/a	Confirmed	
☐	☒	The exact sample size (*n*) for each experimental group/condition, given as a discrete number and unit of measurement
☒	☐	A statement on whether measurements were taken from distinct samples or whether the same sample was measured repeatedly
☐	☒	The statistical test(s) used AND whether they are one- or two-sided<br>*Only common tests should be described solely by name; describe more complex techniques in the Methods section.*
☒	☐	A description of all covariates tested
☐	☒	A description of any assumptions or corrections, such as tests of normality and adjustment for multiple comparisons
☐	☒	A full description of the statistical parameters including central tendency (e.g. means) or other basic estimates (e.g. regression coefficient) AND variation (e.g. standard deviation) or associated estimates of uncertainty (e.g. confidence intervals)
☒	☐	For null hypothesis testing, the test statistic (e.g. *F*, *t*, *r*) with confidence intervals, effect sizes, degrees of freedom and *P* value noted<br>*Give P values as exact values whenever suitable.*
☒	☐	For Bayesian analysis, information on the choice of priors and Markov chain Monte Carlo settings
☐	☒	For hierarchical and complex designs, identification of the appropriate level for tests and full reporting of outcomes
☒	☐	Estimates of effect sizes (e.g. Cohen's *d*, Pearson's *r*), indicating how they were calculated

Our web collection on statistics for biologists contains articles on many of the points above.

Software and code

Policy information about availability of computer code

Data collection	Many of the datasets used are publicly available via the NASA OSDR's Biological Data Management Environment (https://osdr.nasa.gov/bio/repo), including murine skin RNA-Seq datasets (OSD-238, OSD-239, OSD-240, OSD-241, OSD-254), microarray data from the JAXA hair study (OSD-174), and the JAXA CFE data (OSD-530).
Data analysis	Processing scripts used are available via Github {Link to be added prior to publication}.

For manuscripts utilizing custom algorithms or software that are central to the research but not yet described in published literature, software must be made available to editors and reviewers. We strongly encourage code deposition in a community repository (e.g. GitHub). See the Nature Portfolio guidelines for submitting code & software for further information.

Data

Policy information about availability of data

All manuscripts must include a data availability statement. This statement should provide the following information, where applicable:

- Accession codes, unique identifiers, or web links for publicly available datasets
- A description of any restrictions on data availability
- For clinical datasets or third party data, please ensure that the statement adheres to our policy

Many of the datasets used are publicly available via the NASA OSDR's Biological Data Management Environment (https://osdr.nasa.gov/bio/repo), including murine skin RNA-Seq datasets (OSD-238, OSD-239, OSD-240, OSD-241, OSD-254), microarray data from the JAXA hair study (OSD-174), and the JAXA CFE data (OSD-530).

Human research participants

Policy information about studies involving human research participants and Sex and Gender in Research.

Reporting on sex and gender	Inspiration4 (i4) astronaut sample collection<br>Four civilians, two males and two females, spent three days in Low-Earth Orbit (LEO) at 585 km above Earth.
Population characteristics	JAXA astronaut hair follicle data<br>Gene expression data from 10 JAXA astronauts' hair follicles11 was downloaded from the NASA OSDR (OSD-174).<br>JAXA Cell-Free Epigenome (CFE) Study RNA quantification data<br>Aggregated RNA differential expression data and study protocols were shared through the NASA OSDR with accession number: OSD-530<br>RNA-Seq data analysis on Twin Study samples<br>Longitudinal samples were collected from a male astronaut aboard the ISS and his identical twin on Earth during a 340 day mission including 6 months preflight and 6 months postflight follow-up, for a total of 19 timepoints for the flight subject and 13 timepoints for the ground subject.<br>Inspiration4 (i4) astronaut sample collection<br>Four civilians, two males and two females, spent three days in Low-Earth Orbit (LEO) at 585 km above Earth. The mission launched from NASA Kennedy Space Center on September 15th, 2021 and splashed down in the Atlantic Ocean near Cape Canaveral on September 18th, 2021. Several human health and performance related experiments were carried out in collaboration with SpaceX, the Translational Research Institute for Space Health (TRISH) at Baylor College of Medicine (BCM), and Weill Cornell Medicine. Experiments were performed in accordance with the relevant guidelines at the principal investigators' institutions. Moreover, the different study designs and the corresponding methods to collect and analyze the biological samples were approved by BCM IRB.
Recruitment	Inspiration4 (i4) astronaut sample collection<br>Four civilians, two males and two females, spent three days in Low-Earth Orbit (LEO) at 585 km above Earth. The mission launched from NASA Kennedy Space Center on September 15th, 2021 and splashed down in the Atlantic Ocean near Cape Canaveral on September 18th, 2021. Several human health and performance related experiments were carried out in collaboration with SpaceX, the Translational Research Institute for Space Health (TRISH) at Baylor College of Medicine (BCM), and Weill Cornell Medicine. Experiments were performed in accordance with the relevant guidelines at the principal investigators' institutions. Moreover, the different study designs and the corresponding methods to collect and analyze the biological samples were approved by BCM IRB.
Ethics oversight	The different study designs and the corresponding methods to collect and analyze the biological samples were approved by BCM IRB.

Note that full information on the approval of the study protocol must also be provided in the manuscript.

Field-specific reporting

Please select the one below that is the best fit for your research. If you are not sure, read the appropriate sections before making your selection.

☒ Life sciences ☐ Behavioural & social sciences ☐ Ecological, evolutionary & environmental sciences

For a reference copy of the document with all sections, see nature.com/documents/nr-reporting-summary-flat.pdf

Life sciences study design

All studies must disclose on these points even when the disclosure is negative.

Sample size	Sample size was limited due the constraints of sending samples to space. That said, we have the maximum amount of the samples possible to provide best statistics.
Data exclusions	None
Replication	Data from different data sets of mice flown to space and astronauts. Enough replicates per condition exists as outlined in the methods of paper.
Randomization	Not relevant for this study.
Blinding	Blinding not possible due to the conditions of the experiments of samples flown to space.

Reporting for specific materials, systems and methods

We require information from authors about some types of materials, experimental systems and methods used in many studies. Here, indicate whether each material, system or method listed is relevant to your study. If you are not sure if a list item applies to your research, read the appropriate section before selecting a response.

Materials & experimental systems

n/a	Involved in the study
☒	☐ Antibodies
☒	☐ Eukaryotic cell lines
☒	☐ Palaeontology and archaeology
☐	☒ Animals and other organisms
☒	☐ Clinical data
☒	☐ Dual use research of concern

Methods

n/a	Involved in the study
☒	☐ ChIP-seq
☒	☐ Flow cytometry
☒	☐ MRI-based neuroimaging

Animals and other research organisms

Policy information about studies involving animals; ARRIVE guidelines recommended for reporting animal research, and Sex and Gender in Research

Laboratory animals	C57BL/6 and BALB/c
Wild animals	The data was from archived data and not from experiments we performed. All details from the experiments are provided on the NASA GeneLab platform.
Reporting on sex	All mice were Female.
Field-collected samples	The data was from archived data and not from experiments we performed. All details from the experiments are provided on the NASA GeneLab platform.
Ethics oversight	The data was from archived data and not from experiments we performed. All details from the experiments are provided on the NASA GeneLab platform.

Note that full information on the approval of the study protocol must also be provided in the manuscript.
